# Circulation, genomic characteristics, and evolutionary dynamics of class I Newcastle disease virus in China

**DOI:** 10.1080/21505594.2022.2037342

**Published:** 2022-02-21

**Authors:** Lijia Jia, Bilin Liang, Ke Wu, Runkun Wang, Haizhou Liu, Di Liu, Quanjiao Chen

**Affiliations:** a Cas Key Laboratory of Special Pathogens and Biosafety, Wuhan Institute of Virology, Center for Biosafety Mega- Chinese Academy of Sciences, Wuhan, China; b Computational Virology Group, Center for Bacteria and Viruses Resources and Bioinformation, Wuhan Institute of Virology, Chinese Academy of Sciences, Wuhan, China; c Savaid Medical School, University of Chinese Academy of Sciences, Beijing, China

**Keywords:** Newcastle disease virus, genomic epidemiology, genetic evolution, evolutionary dynamics

## Abstract

Newcastle disease caused by Newcastle disease virus (NDV) is one of the most serious threats to chickens and has two clinical forms, typical and atypical, caused by velogenic and lentogenic strains, respectively. To control the epidemic, many vaccines against velogenic class II NDVs have been introduced worldwide, but this has led to accelerated mutation of class II viruses under immune pressure and, on the other hand, to non-vaccine targeting class I NDVs becoming the dominant population in poultry. In this context, this study provided the first large-scale genomic epidemiological and quasispecies dynamic analysis of class I NDVs in China, and found that class I viruses that first appeared in East and South China have spread to central China and become the dominant class with an average evolutionary rate of 1.797 × 10^−3^. In addition, single nucleotide polymorphism and intra-host single nucleotide variation analyses show that HN and P genes have high mutation rates and may act as front-runners for NDV to expand their host range and enhance their virulence. This study also found that the class I NDV population has accumulated a number of mutations under positive selection and that six isolates with shortened C-terminal extensions of the HN protein are evolving toward increased virulence. These results not only enrich the research resources but also help us to better understand the dynamic evolution and mutational trends of NDV at the genomic level, which is crucial for monitoring, early warning, and controlling the outbreak of Newcastle disease.

## Introduction

Newcastle disease (ND) is an acute fever and highly contagious disease caused by the Newcastle disease virus (NDV), which has a high morbidity and mortality and can infect a wide range of birds [1]. It is also a minor zoonosis, and people suffer from conjunctivitis after infection [2]. Since it was first reported from Indonesia in 1926, at least four pandemics have occurred in the world, and each epidemic has been accompanied by the emergence of new genotypes [2–4], causing huge economic losses to the poultry industry.

NDV is a single-stranded, negative-sense RNA virus belonging to the genus *Orthoavulavirus* in the family *Paramyxoviridae* [5]. Its genome is about 15.2kb and encodes six structural proteins: nucleoprotein (NP), phosphoprotein (P), matrix protein (M), fusion protein (F), hemagglutinin–neuraminidase (HN), and large polymerase (L) [6]. The fusion protein is considered to be the most relevant determinant of NDV virulence [7]. NDV only has a single serotype, but it can be divided into two classes based on variable genome size and the diversity of *F* gene, class I and class II [8,9]. According to the new classification system, class I NDV isolated is classified into a single genotype containing three sub-genotypes 1.1.1 (former 1a), 1.1.2 (former 1b), and 1.2 (former 1c and 1d) [10,11]. Most class I NDVs are lentogenic strains that circulate mainly in wild birds and live poultry markets (LPMs) and are often used as vectors for oncolytic viruses or recombinant vaccines [12]. Class II NDVs are more diverse and have been identified to include at least 20 genotypes (I–XX), covering strains of varying virulence from lentogenic to velogenic [10]. The current vaccines are target to class II due to its high pathogenicity to chicken, making class I strains develop rapidly without restrictions, which are prevalent in poultry [13,14]. Although most of the class I strains are lentogenic, studies have shown that the class I virus from waterfowl become virulent after continuous passaging in chicken air sac [15] so the prevalence of class I in poultry remains a potential threat that cannot be ignored.

Similar to the natural reservoirs of the avian influenza virus (AIV), the natural reservoirs of NDV are considered to be wild birds and waterfowls [16]. The continued spread of ND among the population is due to the large number of wild birds and their lack of obvious disease characteristics after being infected with the ND, especially migratory birds, whose migration is a meaningful way for NDV to transmission and communicate across regions [1,17]. The constant threat of virus introduction from wild birds makes biosecurity on poultry farms critical. On the other hand, even after vaccination, multiple genotypes of NDV were still isolated from LPMs [13,14,18]. Antigenic differences between epidemic and vaccine strains have led to the accumulation of atypical ND, which is a challenge to the prevention and control of traditional vaccines.

Therefore, understanding the epidemiological transmission of NDVs in domestic poultry and their evolutionary dynamics can lead to better understanding of the evolution and genetics of the virus characteristics, and early warning of the emergence of new NDV genotypes. Central China is an important stopover site for migratory birds on the East Asian/Australasian flyway, with its abundant bird resources and well-developed poultry industry, making it an ideal site to investigate the communication of wild birds and poultry [19,20]. In this study, we sequenced NDV strains isolated from central China from March 2016 to March 2017 to reveal their genetic and phylogenetic characteristics. In addition, we performed an intra-host variation analysis to characterize and discuss the evolutionary dynamics of the NDV and extant state of its quasispecies [21], providing guidance for epidemiology and prevention of NDV.

## Materials and methods

### Ethics statement

All studies involving animals were carried out in accordance with the animal welfare guidelines of the World Animal Health Organization. With permission of the Monitoring Center of Wildlife Diseases and Resource of Hubei Province, China, migratory bird droppings were collected in the Wang Lake Wetland Reserve and other lakes in Hubei province. Cloacal swabs were collected in LPMs in central China, and there were no special licensing requirements for this activity.

### Sampling and virus isolation

Samples of poultry and wild birds were collected in central China from January 2016 to March 2017. Monthly sampling was conducted in LPMs in Nanchang, Jiangxi province and Yueyang, Hunan province. Swabs were used to collect cloacal swabs and environmental samples including animal drinking water, feed, feces, and slaughter plates in LPMs. Individual fecal samples were collected from Dongting Lake, the Poyang Lake, and lake wetlands in Hubei during migration of wild birds. In addition, tissue samples, such as the lung from a dead swan, were also collected from Longgan Lake in Huangmei County, Hubei province. Each sample was individually inoculated to 10-day-old specific pathogen-free (SPF) chicken embryos for virus isolation. Hemagglutinin (HA)-positive samples were stored at −80°C until use.

### RNA extraction, RT-PCR, and detection

RNA was extracted from the HA-positive allantoic fluids using the Tianlong NP968-C Nucleic Acid Extraction and Purification System with matched EX-RNA/DNA viral nucleic acid extraction kits. Random hexamer primers were used to obtain cDNA, and then NDV Class I/II specific primers were used to perform PCR subtyping (primer sequence: Class I F-CACCAAGCTGGAGAAAGGGCATAC, Class I R-CAGTATGTTTGCAGCATTCTGGTTGG; Class II F-CCATTGCT AAATACAATCCTTTCA, Class II R-CTGCCACTGCTAGTTGTGATAATCC). One hundred and fifty-one NDV-positive RNA samples were qualified for further sequencing using the Agilent 2100 Bioanalyzer System as directed by the manufacturer.

### Next-generation sequencing and genome assembly

Next-generation sequencing (NGS) technology was used to determine the whole-genome sequence of NDV isolates. Each of the 151 NDV isolates was individually barcoded and prepared for sequencing library construction with the NEBNext Ultra II RNA Library Prep Kits. Quality-checked libraries were loaded and sequenced on an Illumina Miseq platform that produced 2 × 150 bp paired-end reads. FastQC (http://www.bioinformatics.babraham.ac.uk/projects/fastqc) was used to detect the quality of raw data. The first 10 low-quality (Q < 20) bases of each read and the residual adapter were filtered and removed by fastp v0.21.0 [22]. Clean reads were *de novo* assembled to obtain consensus sequences using MEGAHIT v1.1.2 [23]. NCBI BLAST+ was used to choose the best matching reference sequences. The insertions and deletions of the UTR region sequences were corrected by aligning them with reference genomes. In order to detect potential recombinant sequences, all NDV sequences were screened using RDP v4 [24] with parameters set to the default, and the recombination was significantly supported when more than three methods showed a Bonferroni corrected *p* value <0.05. The 140 viral sequences isolated in this study were submitted to the GenBank database (accession numbers: MH289831-MH289970).

### Phylogenetic analysis

All available F genes with the complete coding region (1662 nt) and collection information were obtained from GenBank and combined with our data to generate two datasets: class I (n = 537) and class II (n = 2801). Sequences were aligned using MUSCLE v3.8.31 [25] (alignments can be available on request). The maximum-likelihood (ML) phylogenetic analysis was then performed using RAxML-HPC2 v8.2.10 [26] with a GTR-GAMMA model of nucleotide substitution, where branch nodes are supported by 1000 bootstrap replicates.

After removing sequences of samples with an unknown collection date and containing consecutive N bases, two smaller but representative datasets of F genes: class I (n = 505) and class II (n = 1474) were used to construct the Maximum Clade Reliability (MCC) tree based on BEAST v2.6.6 [27]. The base substitution model is the Hasegawa-Kishino-Yano (HKY) + G, and a relaxed molecular clock was used with 500 million iterations. Tracer v1.7.2 [28] was used to confirm the reliability of the results. The tree file was parsed by the TreeAnnotator, and the result was visualized in FigTree v1.4.3 (http://tree.bio.ed.ac.uk/software/figtree).

In addition, the Bayesian skyline plot (BSP) model of BEAST v2.6.6 was used to detect changes in the historical size of the population.

### Geographical reconstruction

After further removing sequences of samples with unknown collection locations, the smaller of the class I dataset (n = 440) was used for discrete trait ancestral reconstruction based on Bayesian algorithm performed by BEAST v2.6.6. After determining the collection time and location corresponding to the sequences, the HKY substitution model under the strict clock was selected with 100 million iterations, and Coalescent Constant Population was used as the prior model. Tracer v1.7.2 was used to read and detect the results of numerical simulations, and all trees were summarized by TreeAnnotator with 10% burn-in cutoffs. The geo-propagation network was visualized by SpreaD3 v0.9.6 [29], and the dynamic process was demonstrated using Google Earth. The final transmission diagram was created with the Adobe Illustrator CC 2019.

### Genome substitutions

Homemade Perl script (https://github.com/zer0liu/bioutils/tree/master/snp) was used to gather statistics of single nucleotide polymorphisms (SNPs). The script takes a given sequence alignment and a TXT file containing coding sequence (CDS) information as input files, counts, and outputs variant site information. The earliest strain of our isolates Environment/CN/JX/13 M/2016 (GenBank accession number: MH289917) was selected as the reference genome. The non-synonymous substitutions, synonymous substitutions, substitutions in non-coding regions, and gaps were marked with different colors by R v4.1.1.

### Intra-host single nucleotide variations (iSNVs)-calling

Clean reads of individuals were mapped to the selected reference genome Environment/CN/JX/13 M/2016 (GenBank accession number: MH289917) using Bowtie2 v2.3.0 [30] and generate “mpileup” files by SAMtools v1.3.2 [31], respectively. A Perl script (http://github.com/generality/iSNV-calling) [21] was used to read mpileup files for iSNV calling. Briefly, first, at each NDV reference genome site, the aligned low-quality sequencing bases (< Q20) and indels were excluded, and the site depth, as well as strand bias was re-calculated. Next, valid iSNVs were called from samples that meet the following criteria: samples with more than 3000 sites with a sequencing depth ≥80× or a minor allele depth ≥5× while the minor allele frequency ≥3%, meanwhile the strand bias of the minor allele was less than tenfold [21].

### Amino acid and potential positive-selection sites analysis

The amino acid sequences of HN, F, and P genes were aligned by BioEdit v7.0.5, and the variation information was displayed via WebLogo [32]. PAML’s codeml program was used to perform site model analysis to detect changes in the selection pressures of HN, F, and P genes during evolution [33]. This study has referred to the following five models for selection calculating: M0 (single ratio model), M1a (neutral model), M2a (selection model), M7 (Beta distribution model), and M8 (Beta-ω distribution model). The χ2 distribution was used to perform a likelihood ratio test (LRT) of the pair null hypothesis and the alternative hypothesis models, M1a and M2a, or M7 and M8, to determine whether to accept an alternative hypothesis based on the *p* value, respectively.

## Results

### Systemic surveillance of NDV in central China

From January 2016 to March 2017, we collected poultry and wild bird fecal samples in five seasons in Hubei, Hunan, and Jiangxi provinces (Table 1). Among the 2619 samples from LPMs in Jiangxi and Hunan, 137 NDV isolates were isolated from chickens, ducks, geese, and pigeons, respectively, with a positivity rate of 5.23%. In contrast, 6852 wild bird fecal samples were collected from Dongting Lake in Hunan province, Poyang Lake in Jiangxi province and Wang Lake in Hubei province. Among them, 14 NDV strains were isolated, with a positive rate of about 0.20%. The distribution of NDV strains in LPMs did not correlate significantly with the month of sampling, while the highest NDV isolation rate in migratory birds was found in February each year (Figure S1). Notably, 57 NDV strains simultaneously tested positive for the AIV (mainly H9N2, H5N6, and H7N9), with a co-infection rate of 37.7%, showing the overlap of transmission routes between the NDV and AIV (Table S1).

**Table 1.** Isolation of NDVs from multiple avian species at LPMs and lake wetlands in China from January 2016 to March 2017

Source	NDV Positive/All Sample					Total	NDV Isolation Rate (%)	Number of obtained NDV genome	Influenza A
	1^st*^ Quarter	2^nd^ Quarter	3^rd^ Quarter	4^th^ Quarter	5^th^ Quarter				Co-infection (No./%)
**Poultry**	32/508	18/365	18/330	26/500	43/916	137/2619	5.23	126	56/44.44
**Wild birds**	13/3620	-	-	0/2195	1/1037	14/6852	0.2	14	1/7.14
**Total**	45/4128	18/365	18/330	26/2695	44/1953	151/9471	1.6	140	57/40.71

*: **1^st^ Quarter refers to January to March 2016, 5^th^ Quarter refers to January to March 2017**

Due to the variation in viral load and genome integrity among the samples, 137 near-full-length genomes, and 3 partial genomes were obtained from 140 samples (14 wild bird-derived and 126 poultry-derived sequences) subjected to NGS sequencing out of 151 total NDV strains detected (Table S1). To avoid laboratory contamination of these sequences, seven algorithms of RDP4 were used to detect them in this study, but the results did not reveal any obvious recombination signal, and there were no suspicious sequences that needed to be rejected.

### Genotyping and phylogenetic analysis of NDV

Multiple sequence comparisons showed that the nucleotide homology of the whole genome among the NDV isolates in this study ranged from 71.0% to 99.9% (Table S2). For the F protein, four cleavage motifs were included, all of which fit the typical profile of lentogenic strains [4,34], with the wild bird-derived ones being dominated by “^112^G-K-Q-G-R-L^116^” (12/14) and the poultry-derived ones by “^112^E-R-Q-E/G-R-L^116^” (116/127) (Table S1, Figure S2). In addition, “^112^G-R-Q-G-R-L^117^” motif, which was different from the two motifs mentioned above, was found in seven chicken-derived NDVs (20C2, 22F2, 31A2, 31J2, 33A2, 37A2, and 38A2). It was found that the two F protein motifs of wild bird-derived NDV (F2B62, F2-95-1) were identical to those of poultry-derived ones, while the F protein motif of four duck-derived (75C2, 77C2, 79C2, 2 N) was identical to that of wild bird-derived ones, suggesting the possible spillover of NDV between poultry and wild birds in the field.

**Figure 1.**
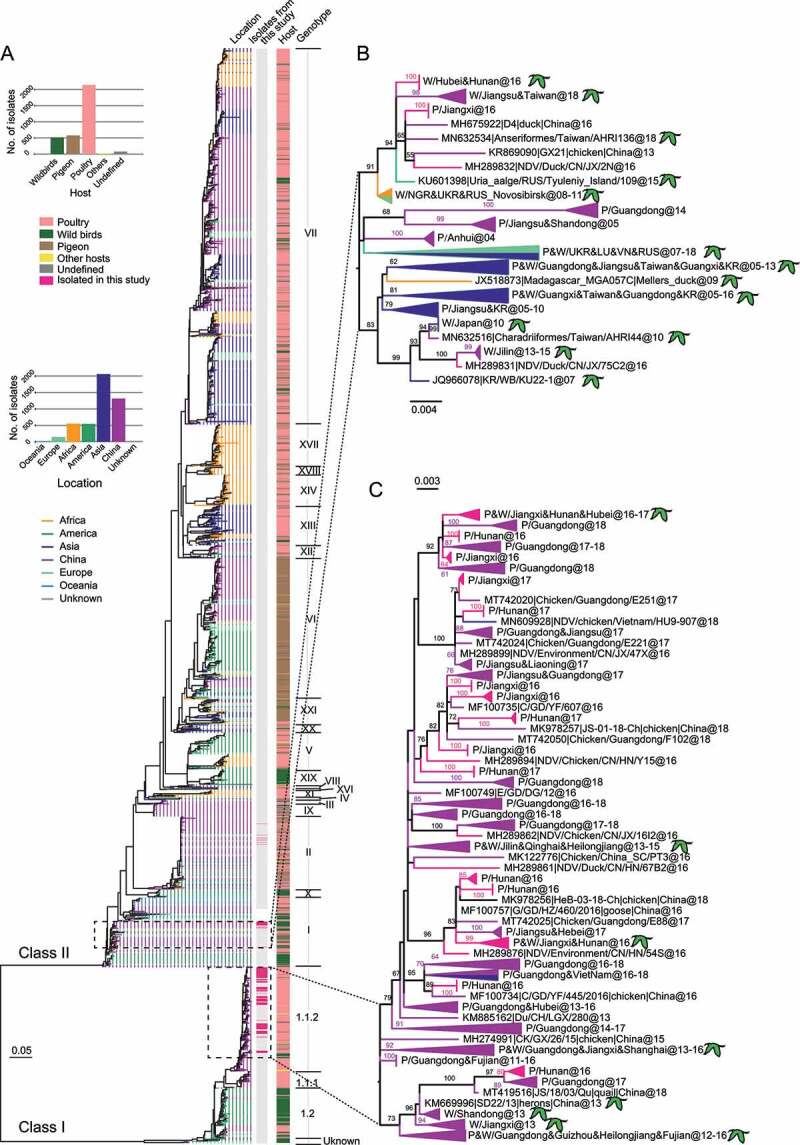
Maximum likelihood phylogenetic tree based on the complete F gene nucleotide sequences. All available GenBank complete F gene (1662 nt) sequences (n = 3338) were used to construct ML tree. The scale bar represents the number of nucleotide substitutions per site. A, Newcastle disease viruses isolated from different geographical regions and hosts are distinguished by colors, meanwhile the strains isolated in this study were marked with a dashed box (vaccine strains not included). B, C, magnification of ML tree of class II genotype I and class I sub-genotype 1.1.2, respectively. The isolates in this study and its’ closer reference strains are highlighted. The branches are colored according to the location on which the virus was isolated. The isolates from wild birds are marked by green birds and only the bootstrap value greater than 60% is shown.

To further trace the genotypes and evolutionary positions, we constructed a maximum likelihood phylogenetic tree by combining the sequences of this study with 3198 F genes in GenBank for which full-length CDS were available. The results of this study showed that three genotypes of NDV were circulating in central China in 2016–2017 (Figure 1(a)). Among them, 117 isolates belonged to sub-genotype 1.1.2 of class I, the nucleotide homology and amino acid homology between these strains are 96.4–99.9% and 98.0–100%, respectively; 16 isolates belonged to genotype I of class II and shared 94.8–95.3% nucleotide homology and 97.2–97.8% amino acid homology with that of Ulster/67 (GenBank accession number: AY562991), an international early isolate of class II genotype I. In addition, seven isolates belonging to genotype II of class II were isolated from farmed chickens, which were similar to the vaccine strain LaSota (GenBank accession number: AF077761) with greater than 99% homology at the amino acid level, so their evolutionary significance will not be discussed here (Table S1).

A phylogenetic tree based on complete F gene sequences showed that the two NDV isolates from wild birds and 115 isolates from poultry in this study clustered together and belonged to class I sub-genotype 1.1.2. The remaining 12 wild bird-derived isolates and 4 poultry-derived isolates gathered and belonged to class II genotype I. A close relationship between NDV isolates poultry and wild birds, revealing a mutual communication between the two hosts. In class II genotype I, isolates from this study clustered with viruses from Tyuleniy Island, Russian Federation (48°30′N, 144°39′E), South Korea (37°33′N, 126°58′E), Japan (35°39′N, 139°44′E), Viet Nam (21°01′N, 105°53′E), northeastern, southern, and eastern China (Figure 1(b)), all of which are located along the East Asian/Australasian flyway. Not only that but they are also genetically close to NDVs from Russian Federation (55°0ʹN, 82°93ʹE), Luxembourg (49°81ʹN, 6°13ʹE), Ukraine (50°28′N, 30°29′E), and Nigeria (9°08ʹN, 8°67ʹE) (Figure 1(b)), which are located on the Black Sea/Mediterranean flyway, constituting a relatively independent branch [35]. Due to its complex and diverse geographical origin, it appears that the evolution and transmission of class II genotype I NDVs is closely related to migratory birds. Likewise, this wild bird-poultry communication was also reflected in class I’s lineage. Class I sub-genotype 1.1.2 NDV consists of isolates from both wild birds and poultry, although poultry unquestionably dominates (Figure 1(c)). Isolates from this study clustered with other Chinese strains but split into two distinct groups, most of which are poultry-derived strains originating from South and East China during 2013–2018.

In addition, to avoid bias caused by evolutionary analysis based on the F-gene tree only, we constructed ML trees based on the whole genome and each structural gene separately (Figure S3). The results show that both the topology and the genotype distribution of different trees are generally consistent with the F-gene tree, indicating that the F-gene-based analysis is representative enough to be trusted.

### Ancestral dating and population fluctuation of NDV

**Figure 2.**
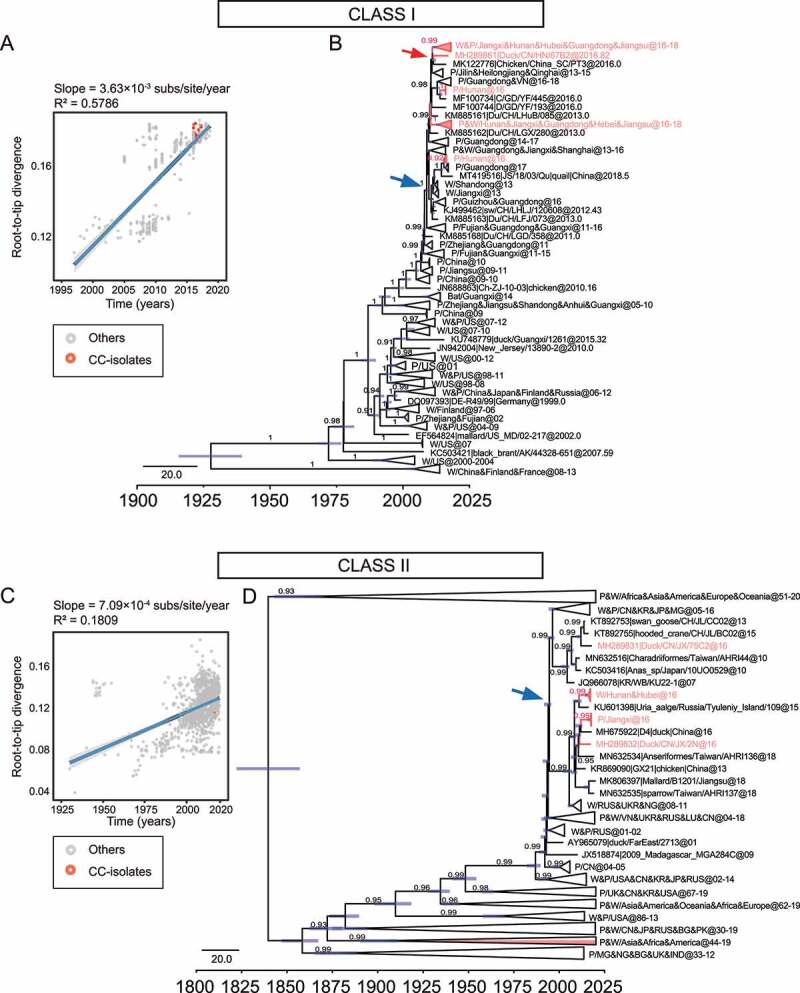
Ancestral dating of class I and class II NDVs a, c, Root-to-tip regression for both class I (A, n = 505) and class II (C, n = 1474) datasets to detect the temporal signals. CC stands for central China; b, d, the MCC tree of class I (b) and class II (d) NDVs. The isolates in this study are marked in rose, and the time of the MRCA of the strains is marked with a rose or blue arrow. P for poultry, W for wild birds.

**Figure 3.**
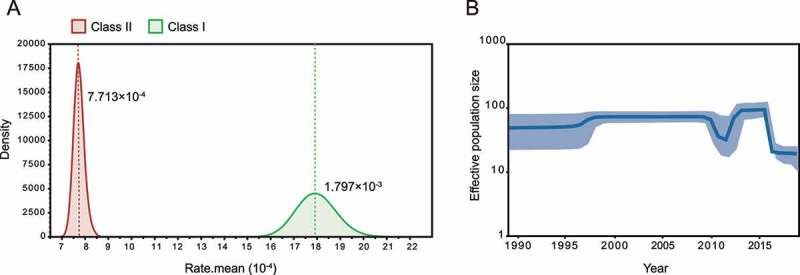
Evolutionary rate and population dynamics of NDV a, Estimated molecular evolutionary rate of NDV; b, Bayesian skyline plot of class I virus, showing effective population size as a function of time. The light blue area represents the 95% confidence intervals of HPD analysis.

Next, we estimate the probable date of arrival of the NDV isolates at the sampling site. Our analysis showed that class I NDVs in central China originated from poultry in South and East China in December 2008 (95% highest posterior density (April 2008, August 2009)), located along the East Asian/Australasian flyway (Figure 2(b), blue arrow). Ancestor NDVs divided into two directions, Jilin/Heilongjiang/Qinghai provinces, and central China, after November 2010 (95% highest posterior density (April 2010, July 2011)) (Figure 2(b), red arrow). Class II genotype I isolates, on the other hand, probably originated from viruses that were endemic in wild birds and poultry in Europe, Asia, and West Africa around July 1994 (95% highest posterior density (September 1991, April 1995)) (Figure 2(d), blue arrow). They share a common ancestor with the earliest European and North American isolates as a result of the evolution of NDV in wild birds in a long-term cycle with an average evolutionary rate of 7.713 × 10^−4^ substitution/site/year (Figure 3(a)).

The class I dataset has a stronger temporal signal compared to the class II NDV (Figure 2(a-c)), which emerged later but evolved faster at 1.797 × 10^−3^ substitution/site/year (Figure 3(a)), indicating the potential threat of lentogenic strains as mutation reservoirs. Therefore, we further investigated the population dynamics of class I NDVs in China. BSP analysis showed that the population size of the class I NDV was stable until 2010, with a brief decline in 2010 followed by a re-expansion in 2012 and then continued to remain stable; after 2016, the effective population size continued to decline, and low levels of genetic diversity were shown among sequences (Figure 3(b)). It seems that class I virus has reached a state of stable adaptation to poultry; however, in addition to this the sample collection information is limited, and the isolates from the same place in this study have a high similarity may also be responsible for the low genetic diversity.

### Circulation and possible transmission route of class I NDV

**Figure 4.**
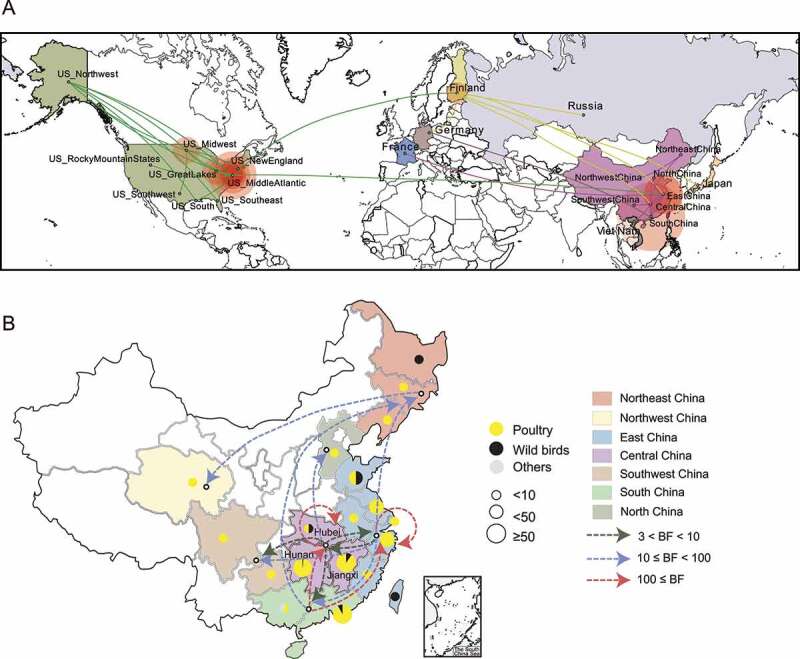
Estimated transmission route of class I NDV Transmission map was drawn based on available NDV with complete sampling information. a, Routes of class I NDV transmission worldwide. The green, lemon-yellow and purple lines represent the transmission from the United States, Finland and China respectively; b, Circulation of NDVs across geographic subdivisions of China rather than between provinces and cities. The number of isolates and the host information are represented as pie charts, and the different colors of the dashed lines represent the values of Bayes factor (BF).

To trace the origin of the ancestors of the class I NDVs, we reconstructed their geographic status. The MCC tree showed that NDVs isolated from North America clustered at the root of the phylogenetic tree, while isolates from Europe and Asia clustered in a lineage and share a common ancestor (Figure S4). Based on the timescale calculated from the available sequence information, NDVs from North America first circulated to European regions such as Finland and Germany, and then spread to Asia and adjacent regions around 1992 (Figure 4(a)).

For mainland China, the class I virus first appeared in poultry in Jiangsu province, then spread to the eastern coastal region via Zhejiang province, and was restricted to eastern China only in the early days; after 2009 class I virus did start to spread widely in circulation (Figure S4). Class I NDVs carried by both poultry and wild birds were present in East China; among them, poultry-derived virus spread to poultry in south and southwest China, and to wild birds in east and northeast China (Figure 4(b)). NDV in East and South China then spread to central China, where transmission of poultry-to-poultry and poultry-to-wild birds occurred. The chaotic interaction of NDVs with poultry and wild birds in east, south, and central China caused class I NDV to spread further and eventually reach the northwest and northeast regions (Figure 4(b)). Our findings suggest that frequent cross-provincial trade in live birds is a major consideration for inter-poultry transmission, while wild birds play an important role in the introduction of viruses to new areas, but where and when this introduction occurs remain uncertain due to limited sampling data.

### Genomic diversity of class I NDV

**Figure 5.**
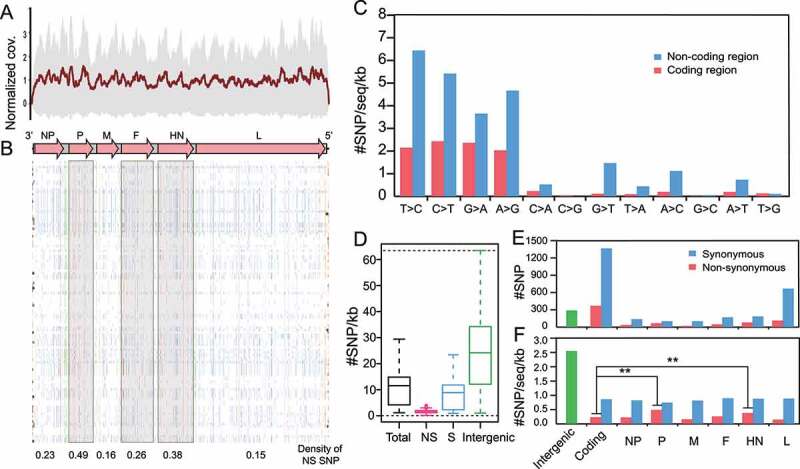
Genomic variations of the class I NDV a, Average sequencing depth of class I NDV samples (ordinate scaled by 600x); b, SNP distributions in class I NDV genomes: magenta for synonymous (s), dark blue for non-synonymous (NS), gray for UTR, and cyan for IG; c-e, Types and distribution statistics of SNPs.

Genomic variations are associated with potential functional changes in the NDV. We analyzed the SNP of class I NDVs. Raw reads of 114 class I NDVs were mapped to the reference genome 13 M (GenBank accession number: MH289917, the earliest class I isolates in this study) with a normalized average coverage of approximately 600 x (Figure 5(a)), satisfying the sequencing depth condition for SNP extraction. We obtained 2127 SNPs throughout the dataset, of which 1736 SNPs are located in the CDS region, and 391 in the intergenic (IG) region and the untranslated region (UTR), with most SNPs being synonymous substitutions (Figure 5(b)). In terms of nonsynonymous substitutions, the top three genes with the highest density of NS SNPs were P, HN, and F (Figure 5(b)).

Furthermore, we tested the mutation types of each site, showing that transitions accounted for about 89% of the total substitutions and accumulated more in the non-coding regions (Figure 5(c)). Most mutations are synonymous with substitutions (Figure 5(d,e)), implying that non-synonymous substitutions may be deleterious and thus purified during evolution, and class I virus appeared to have adapted stably to the host after long-term selection. Consistent with expectations, after normalizing the SNP distribution according to genome number and length of each region, IG became the most SNP-rich region (Figure 5(d-f)), suggesting class I NDVs remained under negative selection overall. However, it is worth noting that HN and P genes had significantly higher rates of non-synonymous substitutions than other structural genes and may play a more important role in adaptation to the host (two-tailed Fisher’s exact test for a 2 × 2 contingency table, *P* = 7.74 × 10^−3^ and 7.67 × 10^−5^, respectively).

### Intra-host evolutionary dynamics of class I NDV

**Figure 6.**
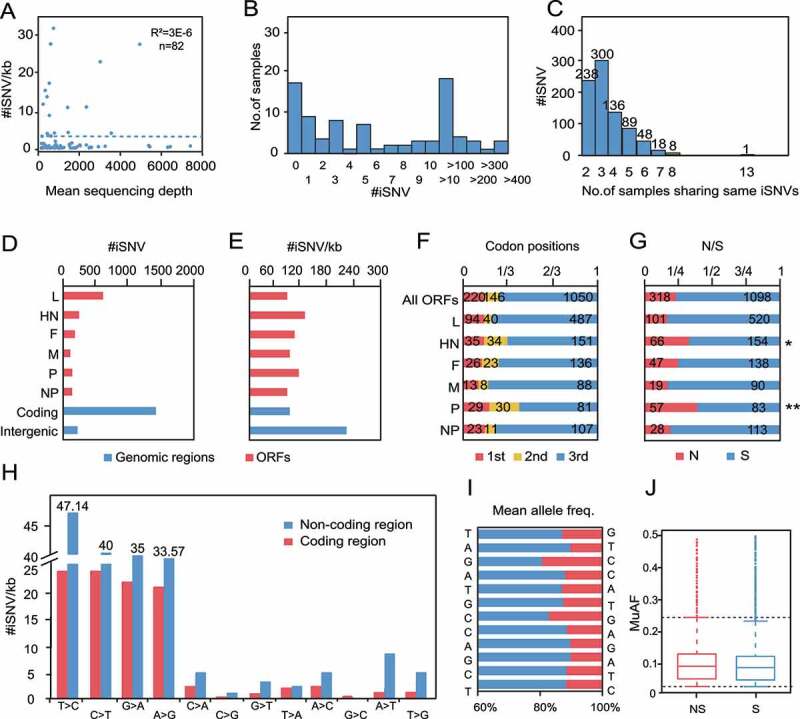
iSNV distributions along the genome of class I NDV a, #iSNV/kb versus mean sequencing depth for each site over NDV genomes. Dashed line shows the linear regression; b, Histogram of iSNVs; c, Statistics of shared iSNV sites; d,e, Total iSNV numbers (d), and #iSNV/kb values (e) along NDV genomic regions and ORF regions; f,g, Distributions of iSNV sites at codon positons (f), and type of iSNVs in each ORF (g). The observed numbers for each ORF are compared with all ORFs (two-tailed Fisher’s exact test for a 2 × 3 or one-tailed 2 × 2 contingency table); h, The variation type of substitutions in coding and non-coding regions; I-J, Mean allele frequency of each substitution.

We next observed the intra-host variation characteristics of class I NDVs at the quasispecies level, and a total of 1678 iSNVs were detected in the 65 samples that met the iSNV calling criteria. To determine whether the quantity of NGS data affects iSNV-calling, we tested the correlation between iSNV/kb (the number of iSNVs per kb) and the mean sequencing depth, which showed no correlation (Figure 6(a)), suggesting the bias caused by NGS in the data is basically negligible, and iSNV/kb could be used to measure the differences between samples or genomes. Most samples contained iSNVs under 100 (Figure 6(b)), and interestingly, 837 iSNVs were found in multiple samples (Figure 6(c)), hinting a correlation with transmission among hosts.

Theoretically, if NDVs evolve under neutral selection, the distribution of iSNVs is random, and the ratio of synonymous to non-synonymous substitutions is similar in all regions. In this study, 85% of the iSNVs were concentrated in the CDS region, while the IG region has the highest density of iSNVs (Figure 6(d,e)). Each open reading frame (ORF) of class I NDV was under negative selection, but the HN and P genes showed more iSNVs on the second codon than the others (two-tailed Fisher’s exact test for a 2 × 3 contingency table, *P* = 0.02, 1.67 × 10^−5^, respectively) and had a significantly higher rate of non-synonymous than the others (one-tailed Fisher’s exact test for a 2 × 2 contingency table, *P* = 0.01, 3.60 × 10^−6^, respectively), consistent with the results for SNPs (Figure 6(f,g)). This suggests that HN and P may have undergone more positive selection. In addition, each iSNV extracted in this study was a two-base mixture and no 3- or 4-base polymorphisms were found, so transition remained the main type of mutation, with a very high accumulation of T-to-C mutations in the non-coding region (Figure 6(h)). The major allele frequency was about 87.00 ± 7.00%, and both synonymous and non-synonymous substitutions showed a high proportion of low-frequency alleles (MuAF <0.1), suggesting a strong purification effect, and the adaptive evolution of the class I NDV has stabilized (Figure 6(i,j)).

### Amino acid analysis of surface antigen genes

The HN and F proteins, the two surface antigens of NDV exhibit the results of immune pressure more visually than other structural proteins, so we observed the diversity of amino acid sequences and the selection pressure to which they were subjected. Using the codeML method of PAML [33], the dN/dS of the HN and F proteins of class I NDV were calculated to be 0.1501 and 0.1627, respectively, which are much smaller than the neutral selection ratio of 1, indicating that the overall evolution was driven by strong purification. However, some potential positive selection sites exist (Table 2).

**Table 2.** Potential positive selection sites for HN and F proteins under multiple models

Gene	dN/dS (M0)	M1 VS. M2		M7 VS. M8		Positive selection sites
		2ΔlnL	*p* value	2ΔlnL	*p* value	
HN	0.1501	2E-05	0.9999	9.3072	0.0095	32 V,293 G
F	0.1627	4.9574	0.0839	37.4233	7E-09	6S,8 G,9Y,11 H,13 F,18 V,21S,23 V,24 F,27 G,115E,337 H,402A,422 K

Using Naive Empirical Bayes (NEB) analysis, the valine at position 32 and glycine at position 293 of the HN protein were under positive selection pressure (Figure S5A, B). The amino acids region at positions 234–329 is involved in receptor binding on the host cell membrane, so it is high that the mutation at position 293 will be retained under positive selection and affect the function of the virus-host binding. On the other hand, it has been suggested that the terminator position of HN is evolutionary and genotype dependent, with most HN lengths at 571 or 577 aa, whereas in lentogenic NDVs such as Ulster/67, HN is 616 aa and exists as an inactive precursor to the HN protein, HN0 [36]. The extra 45 aa extensions at the C-terminus makes HN adsorption activity reduced and puts NDV in a self-repressed state. In this study, the HN of class II genotype II NDVs was 1734 nt long and encoded 577 aa, a normally active HN protein; Class II genotype I strains and most class I sub-genotype 1.1.2 strains had a 45 aa extension at the C-terminus of the HN protein, which was 616 aa long; in addition to this, there were six class I isolates with HN length shortened (HN encoded 612 aa in 92 N and 94 N, 585 aa in 19B2, 24B2, 27B2, 31B2) (Figure S5A), and the activity was between HN0 and HN, showing a tendency to evolve toward enhanced virulence.

The F protein mediates the membrane fusion of NDV with the host cell, which in turn leads to the entry of viral genetic material, and is a key protein associated with NDV virulence. Its ORF is 1662 nt and encodes 553 amino acids. As described above, the F protein cleavage motif of class I NDV in this study is ^112^E-R-Q-E/G-R-L^117^ (Figure S2), where the glycine at positions 112 and 117 was replaced by glutamic acid compared to the classical vaccine strain LaSota (^112^G-R-Q-G-R-L^117^). F0 proteins with this sequence feature are not easily cleaved by proteases and are generally packaged into nascent viruses in an inactive form, resulting in NDV particles with no membrane fusion activity and low infectivity. Selection pressure analysis of the amino acid sequence of the F protein showed that the sites under positive selection pressure were concentrated in the N-terminal signal peptide region of the F protein (1–32 aa) (Figure S5C), but this region is not usually considered to be a functional region; in addition to this amino acid at positions 115, 337, 402, and 422 were also calculated as positive selected sites, with amino acids at positions 337, 402, and 422 are located in the head of the F protein (Figure S5D), while the cleavage site at position 115 is directly associated with NDV virulence.

It is worth stating that, considering that P gene is another structural gene with a high mutation rate in this study, we also analyzed its amino acid sequence, but neither NEB nor Bayes Empirical Bayes (BEB) analysis identified a potentially positive selected site for P protein.

## Discussion

Newcastle disease is currently well controlled in Canada, the United States, and some western European countries, and has been weakened into a small endemic epidemic in parts of Africa, Asia, and South America [2]. However, due to the characteristic of wild birds carrying NDVs without disease, the threat of outbreaks following contact with wild birds exists in any area where poultry is kept, and long-term continuous epidemiological surveillance of NDV is necessary [4]. Although NDV was discovered as early as 1926, research on it has long been focused on velogenic strains, with limited research on lentogenic strains, and only 49 genome sequences of class I NDV were available in GenBank as of the start of this study. In this study, 140 NDV genomes were isolated from continuous virus surveillance in wild birds and poultry in central China, among which 117 strains belonged to class I, providing the necessary basic data for in-depth analysis of the molecular characteristics and evolutionary dynamics of class I NDV.

Phylogenetic analyses showed that NDVs isolated from different regions of the world are highly homologous [3] and that migratory birds are the most likely cause of NDV transmission across geographic areas and play an important-bridging role in the evolution of the NDV cycle. The NDVs isolated from wild birds and poultry in this study had high homology and similar sequence motifs, showing virus spillover between different hosts. In addition to clustering with NDVs on East Asia-Australia and Central Asia flyways, class II NDVs from central China were also genetically close to isolates from Nigeria and Luxembourg on the Black Sea/Mediterranean flyway. Although the Black Sea/Mediterranean flyway does not pass through China, the three flyways intersect in Siberia, and virus transmission may occur there. However, due to the lack of sufficient sequence information, the clear transmission route of NDV worldwide cannot be restored yet. On the other hand, poultry transport may be responsible for the prevalence of class I NDV in domestic poultry. The complex poultry breeding environment in China makes wild birds and domestic waterfowls have a shared ecological interface, and many retail farmers in central China free-range their poultry, especially in the Dongting Lake area with a “poultry-water body-migratory birds” breeding model. Migratory birds and poultry can exchange the virus through direct contact or contaminated water and feed, opening a channel for the communication of NDV between natural hosts and susceptible hosts. In this case, standardized management of retail farming is the most direct and basic preventive and control measure, which can limit the contact between wild birds and poultry, cut off the transmission chain and facilitate the implementation of a unified immunization program, thus enhancing the protection of poultry. It is also important to note that the co-infection of NDV and AIV would make the traditional clinical diagnosis difficult or even have a disease enhancing effect on each other [37], and it is necessary to popularize rapid laboratory diagnosis methods among farms and individual farmers in order to respond to the epidemic and prevent treatment correctly and timely.

All NDV isolates in this study are lentogenic strains, including genotypes of class I and class II. Class I NDVs were reported in 2003 later than that of class II (in 1926) [12]. It is generally accepted that they all originated in wild waterbirds as lentogenic viruses, with the class II NDV adapting earlier to land birds and evolving a large number of velogenic strains [38,39], so class I viruses may have the same evolutionary path. Since the current vaccine only targets class II strains, which allows unprecedented development of class I NDV (with a detection rate of approximately 5.23% in poultry and an evolutionary rate of 1.797 × 10^−3^ substitution/site/year). Poultry carrying the virus, serving as a mobile mutational pool for NDV, makes outbreaks of new genotypes more likely. Previous studies have shown that 1.1.2 is the prevalent sub-genotype of poultry in East China [14,40] and South China [41] since 2008, and the present study further confirmed that 1.1.2 is also the dominant sub-genotype in central China. The most recent common ancestor of NDV in central China originated in East and South China around 2008, indicating that class I NDV has spilled over from the epicenter to the periphery and become the dominant genotype of LPMs in China. In this context, it is important to strengthen LPM regulation and biosecurity awareness for a good and sustainable development of the poultry industry.

Quasispecies dynamics is the main biological feature that decisively influences viral behavior, and its structure is related to the evolution of viruses within and between hosts [42]. The existence of NDV quasispecies was first recognized by Granoff in the 1960s in his study on the transformation of plaques in mixed NDV infections [43]; The availability of high-throughput sequencing technologies has allowed scientists to study quasispecies at the large-scale population levels, such as the resolution that 26% of the quasispecies of the lentogenic Australian NDV strain PR-32 consisted of velogenic NDVs [44]. It was also observed that NDV pathogenesis is regulated by the ratio of lentogenic and velogenic genomes and their interactions, demonstrating for the first time that NDV quasispecies diversity can determine the pathogenic potential of viral populations [15]. Thus, investigating the quasispecies characteristics of NDV are an important way to clarify its pathogenicity and evaluate its threat to poultry industry. Our results indicate that class I NDV is currently circulating in a lentogenic form; however, the dynamics of the quasispecies suggest that the HN and P genes are the genes that accumulate the most variation under host-virus interactions. Moreover, the sequence length and potential positive selected sites of the HN protein showed a tendency for NDV to have variable activity (Figure S5A) [45]. Considering that the HN protein is involved in receptor recognition, and interacts with F proteins to promote specific membrane fusion, its non-conservative nature may lead to the emergence of different antigens during adaptation to the host [3]. The P protein is required for viral RNA synthesis and generally functions in complex with the L protein in viral replication and transcription, and it has been shown that the activity of this complex is directly related to the NDV virulence [46,47]. Thus, the accumulation of variants in the P gene may represent the result of a mutual check and balance between the host immune system and NDV replication. The evolutionary path of RNA viruses is determined by natural selection and is also subject to stochastic effects of genetic drift, mutation, and recombination [48]. RNA viruses undergo frequent population changes as they pass from host to host, so understanding the evolutionary drivers and characteristics of viral populations is essential for evolutionary prediction. In general, class I NDVs are still subject to negative selection; however, some accumulation of variants driven by positive selection were found in this study so in future surveillance programs we should not only focus on the high variable region of the F gene but other genes, especially the HN and P genes need to be focused on.

In summary, this study explored the prevalence of NDV in central China and found that the average evolutionary rate of isolation was bounded between 1.797 × 10^−3^ and 7.713 × 10^−4^ substitution/site/year and there was poultry-wild bird cross-host transmission. Circulating between poultry and wild birds can accelerate the evolution of virulence in class I NDV, which may progressively mutate to strong virulence and lead to outbreaks of new genotypes of ND, jeopardizing the farming economy, so we suggest regulating the use of vaccines, developing new genotypes of vaccines and avoiding cross-farming environments. In addition, we found that the HN and P genes of the dominant sub-genotype 1.1.2 have a higher degree of non-conservativeness and may play a role in acquiring virulence under-altered host environments or other pressures. Future ongoing monitoring of NDV in live poultry markets and wild birds in multiple regions is needed to improve our understanding of class I viruses and to prevent and control outbreaks of new genotypes of ND.

## Data Availability

The accession numbers for the 140 viral sequences isolated in this study is submitted to the GenBank database (accession numbers: MH289831-MH289970). Sequence alignments and other original data are available from the corresponding author on request. The supplementary files were submitted to the FigShare website with a DOI of 10.6084/m9.figshare.18143093 (https://figshare.com/s/8c19e3ed394342106e7a).
